# Effect of Cooking on Protein Digestion and Antioxidant Activity of Different Legume Pastes

**DOI:** 10.3390/foods10010047

**Published:** 2020-12-26

**Authors:** Marta Gallego, Milagros Arnal, José Manuel Barat, Pau Talens

**Affiliations:** Departamento de Tecnología de Alimentos, Universitat Politècnica de València, Camino de Vera s/n, 46022 Valencia, Spain; margalib@upv.es (M.G.); miarsa@upv.es (M.A.); jmbarat@tal.upv.es (J.M.B.)

**Keywords:** legumes, heating, rheology, digestibility, proteolysis, antioxidant peptides

## Abstract

Legumes are protein-rich foods that can be used to prepare pastes or pureed foods suitable for babies and the elderly. The aims of this study were the characterization of different legume pastes (from soybean, lentil, and pea) subjected to three processing methods (ordinary cooking, pressure cooking, and microwave) and the evaluation of protein digestion and antioxidant activity during simulated gastrointestinal digestion (GID). The different cooking methods of legumes led to differences in the physicochemical properties of the pastes, as well as on the textural and viscoelastic characteristics, except for soybean samples, despite all the pastes presenting elastic properties and weak gel behavior. Cooking followed by GID improved the protein digestibility and antioxidant activity of the legumes, which was attributed to released peptides and amino acids more than free phenolics. However, the fate and extent at each digestion stage was different according to the legume type and cooking method, as it would be influenced by the matrix structure and interaction between components. This work has expanded knowledge about the properties, digestibility, and antioxidant activity of different cooked legumes for a future design of pastes.

## 1. Introduction

Legumes are consumed worldwide because of their good nutritional value. They are a rich source of proteins and provide significant amounts of dietary fiber and resistant starch and low amounts of fat; they also offer many essential vitamins and minerals. In fact, legumes represent the major source of vegetable proteins, ranging from 20 to 40%. Lupins and soybeans present the highest protein content, followed by groundnuts, beans, broad beans, lentils, vetches, chickpeas, and peas [1,2]. Legumes can be used, among others, as ingredient in salads or to make soups, stews, pastes, and purees. In this regard, legume pastes and purees can be suitable to prepare foods for babies, as well as for the elderly or people with chewing or swallowing difficulties [3,4]. Nevertheless, their low protein digestibility, low amounts of sulfur-containing amino acids, and the presence of anti-nutritional compounds (ANC) keep their use for human nutrition below their total nutritional potential [5].

Many studies have been carried out in order to evaluate how the nutritional quality of legumes can be improved by the application of processing methods, such as soaking or cooking, which would enhance digestion and absorption of proteins mainly through the inactivation or removal of ANC [6,7,8]. However, the bioavailability of nutrients is determined by the action of enzymes during gastrointestinal digestion, which could also involve the release of bioactive compounds [8,9]. The nutritional characteristics of legume seeds have been associated with exerting beneficial health effects by controlling and preventing metabolic diseases, such as type II diabetes, high cholesterol levels, heart diseases, and cancer [10]. Indeed, many of these effects have been attributed to the antioxidant properties provided by bioactive peptides and phenolic compounds [11,12].

The availability and bioactivity of proteins are primarily determined by their physicochemical properties and amino acid composition and structure, which in turn would depend on the protein source and which can be modified by the applied food-processing conditions. To date, information on the fate and extent of protein digestibility and antioxidant activity of legumes throughout gastrointestinal digestion is still limited. Thus, the aims of this study were the characterization of different legume pastes (from soybean, lentil, and pea) subjected to three processing methods (ordinary cooking, pressure cooking, and microwave), through the study of their physicochemical, textural, and viscoelastic properties, as well as the evaluation of protein digestion and antioxidant activity during simulated gastrointestinal digestion.

## 2. Materials and Methods

### 2.1. Chemicals and Reagents

Enzymes α-amylase from human saliva, pepsin from porcine gastric mucosa, and pancreatin from porcine pancreas, as well as bile extract porcine, trinitrobenzenesulfonic acid (TNBS), 2,2-diphenyl-1-picrylhydrazyl (DPPH), butylated hydroxytoluene (BHT), potassium persulfate, and 2,2′-azino-bis(3-ethylbenzothiazoline-6-sulfonic acid) diammonium salt (ABTS), were purchased from Sigma-Aldrich, Co. (St. Louis, MO, USA). Potassium ferricyanide and ferric chloride were acquired from MP Biomedicals, LLC. (Solon, OH, USA), and trolox was purchased from Acros Organics (Fair Lawn, NJ, USA). Trichloroacetic acid (TCA), ascorbic acid, Folin–Ciocalteu reagent and gallic acid were purchased from Scharlau Chemie, S.A. (Sentmenat, Barcelona, Spain). All other reagents and chemicals were of analytical grade.

### 2.2. Preparation of Legume Pastes

Soybean (*Glycine max*), lentil (*Lens culinaris*), and pea (*Pisum sativum*) seeds were purchased from a supermarket (Madrid, Spain). Seeds were washed and steeped overnight, in tap water, at a legume:water ratio of 1:3 (*w*/*v*), to simulate domestic preparation. Then, the soaked water was drained off, and the seeds were cooked in water at a legume:water ratio of 1:6 (*w*/*v*), by three different household processing methods: (a) ordinary cooking, at 100 °C for 40 min; (b) pressure cooking, at 8.7 psi for 15 min; and (c) microwave cooking, at 800 W for 30 min, using a domestic microwave oven (model MWE 250 FI, Teka, Germany). A total of 200 g of each cooked legume was mixed with 30 mL of water and then homogenized by grinding with a blender for 3 min, until having a smooth paste. Two independent batches were performed for each sample. Samples were named as the type of legume, namely SO (soybean pastes), LE (lentil pastes), and PE (pea pastes), followed by a letter indicating the applied cooking method: C (ordinary cooking), P (pressure cooking), and M (microwave cooking).

### 2.3. Characterization of Legume Pastes

#### 2.3.1. Physicochemical Parameters

The legume pastes were analyzed in triplicate for their physicochemical properties: moisture content, water activity, pH, soluble solid content, and color.

The moisture content of samples was determined by drying them in an oven, until getting a constant weight, and the calculated as follows: Moisture (%) = (W2 − W3)/(W2 − W1) × 100, where W1 is the weight of empty crucible, W2 is the weight of crucible and sample before drying, and W3 is the weight of crucible and sample after drying [13].

The water activity (a_w_) was determined at 25 °C, using an AquaLab series 3 water-activity meter (Decagon Devices, Inc., Pullman, WA, USA). Saturated saline solution was used for calibration of the equipment.

The pH was measured by using a pH-meter (Basic 20+, Crison Instruments, S.A., Barcelona, Spain), at room temperature.

The total soluble solids (° Brix) were measured by using a digital refractometer (model HI 96801, Hanna instruments, Woonsocket, RI, USA).

The color was determined by using a Minolta CM-700d spectrophotometer (Konica Minolta Sensing, Inc., Osaka, Japan). Samples were placed on a white standard plate, and the standard light source D65 and standard observer 10° were used to obtain color coordinates L^*^ (lightness), a* (redness), and b* (yellowness), as well as C* (chroma, calculated as (a*^2^ + b*^2^)^1/2^) and h^*^ (hue angle, calculated as arctg(b*/a*)).

#### 2.3.2. Texture and Viscoelastic Behavior

The texture of the legume pastes was determined by their extrusion properties, using a TA-TX2 texture analyzer (Stable Micro Systems Ltd., Reading, UK). Back-extrusion test was performed by using a compression probe (35 mm of diameter) that was driven into a bucket (measuring 50 mm in diameter and 70 mm in height), to compress the sample. The measuring cup was filled with the sample, up to a height of 40 mm, and the test was carried out at 1 mm s^−1^ compression rate, up to 75 % sample strain. The maximum force (firmness) and area under the curve (AUC; consistency) were obtained from the force–distance profiles, using the Exponent software (Stable Micro Systems Ltd.). Data were obtained from two independent experiments for each cooked legume.

The viscoelastic characterization of the legume pastes was carried out in a Kinexus Pro+ Rheometer (Malvern Instruments Ltd., MA, USA) equipped with a parallel plate geometry (PLC61/PU40), according to Talens et al. [14], but with some modifications. A large amplitude oscillatory stress shear (LAOS) test was conducted to characterize the non-linear viscoelastic properties and to determine the limits of the linear viscoelastic region (LVR) and the flow point of the samples. The stress sweep test was performed at 1 Hz within a range from 1 to 2000 Pa. The changes in elastic modulus (G’, Pa) and viscous modulus (G’’, Pa) with stress, as well as the elastic modulus value at LVR (G’_LVR_, Pa), the stress value at LVR (Stress_LVR_, Pa), and the flow point (Pa) were recorded. The linear viscoelastic properties of the samples were also characterized by a small amplitude oscillatory shear (SAOS) test, which was run from 0.1 to 100 Hz within the LVR. The changes in elastic modulus (G’, Pa), viscous modulus (G’’, Pa), complex modulus (G*, Pa), complex viscosity (η*, Pa), phase angle (δ, °), and loss tangent (Tan δ = G”/G’, dimensionless) were recorded. Data were obtained from two independent experiments for each cooked legume.

### 2.4. In Vitro Gastrointestinal Digestion

In vitro gastrointestinal digestion (GID) was performed by following the standardized INFOGEST method [15]. The preparation of digestive fluids (simulated salivary fluid (SSF), simulated gastric fluid (SGF), and simulated intestinal fluid (SIF)) and the determination of enzyme activities were also carried out according to Minekus et al. [15].

The in vitro GID was performed in 1 g of each sample, in duplicate. The oral phase was simulated by adding SSF-containing (pH 7) salivary α-amylase (75 U/mL) to the sample in a ratio of 1:1 (v/w). The mixture was flipped from top to bottom, at 40 rpm, for 2 min, at 37 °C, using an Intell-Mixer^TM^ RM-2 (ELMI Ltd., Riga, Latvia) and an incubator chamber Selecta (JP Selecta, S.A., Barcelona, Spain). In the gastric stage, the oral bolus was diluted 1:1 (*v*/*v*) with SGF and gastric enzyme pepsin (2000 U/mL). The pH was adjusted to 3 with HCl (1 M), and the mixture was incubated under agitation for 2 h at 37 °C, as previously described. The intestinal phase was simulated by diluting the gastric chyme with SIF-containing (pH 7) bile salts (10 mM) and pancreatin (based on trypsin activity to achieve 100 U/mL), in a ratio 1:1 (*v*/*v*). The pH was adjusted to 7 with NaOH (1 M), and the sample was incubated for 2 h at 37 °C as described above. Enzymatic reactions were stopped by heating (100 °C, 5 min), and then the samples were immediately placed in ice. Separate tubes were used for each sampling time point (after oral, gastric, and intestinal phases), and samples before digestion were obtained by mixing the legume paste with water in a ratio 1:1 (*w*/*v*). All the samples were finally centrifuged (8000 g, 4 °C, and 10 min), and the resultant supernatants were taken and stored at −20 °C, for subsequent analyses of protein digestibility, free phenolics, and antioxidant activities. Blank samples containing all enzymes and bile (without sample) were considered and tested in the following assays, subtracting the obtained values from the results of samples.

### 2.5. Total Soluble Proteins, TCA-Soluble Peptides, and Free Amino Groups

The content of total soluble proteins was determined according to the Bradford assay [16]. In triplicate, 40 μL of sample was mixed with 2 mL of Bradford reagent and incubated for 5 min, at room temperature, before measuring the absorbance at 595 nm, using a UV–Visible spectrophotometer (Helios Zeta, Thermo Scientific, UK). Results were expressed as mg bovine serum albumin (BSA) protein/g sample (dry weight, dw).

The content of TCA-soluble peptides was determined based on the methodology used by Ketnawa et al. [17]. A total of 50 μL of each sample, in triplicate, was added to 450 μL of TCA (5%, *w*/*v*), vortexed, and kept at 4 °C for 1 h. After centrifugation (8000 g, 10 min), the supernatant was taken, and the TCA-soluble peptide content was determined by measuring the absorbance at 280 nm. Results were expressed as mg tyrosine equivalents/g sample (dw).

The content of free amino groups was determined by using the TNBS method [18]. Briefly, 40 µL of each sample, in triplicate, was mixed with 320 µL of sodium phosphate buffer (0.2 M, pH 8.2) and 320 µL of TNBS (0.1 %, v/v), shaken, and heated at 50 °C for 60 min. Then, 640 µL of HC1 (0.1 N) was added, and the absorbance was measured at 340 nm after 30 min of incubation. Results were expressed as mg L-leucine equivalents/g sample (dw).

### 2.6. Total Free Phenolics

The content of total free phenolics was determined according to Donlao and Ogawa [19]. In triplicate, 64 μL of the sample was mixed with 320 μL of Folin–Ciocalteu reagent (10%, *w*/*v*) and 256 μL of Na_2_CO_3_ (7.5%, *w*/*v*). The mixture was incubated for 1 h, at room temperature, and then the absorbance was measured at 765 nm. Results were expressed as mg gallic acid equivalents/g sample (dw).

### 2.7. Antioxidant Activity

#### 2.7.1. DPPH Radical Scavenging Activity

The DPPH activity was determined by following the methodology described by Bersuder, Hole, and Smith [20]. For that, 80 μL of each sample was mixed with 400 μL of ethanol and 100 μL of DPPH solution (0.02%, *w*/*v*) and incubated in the dark for 1 h. The reduction of DPPH^•^ was measured at 517 nm, and BHT was used as a positive control. Analyses were performed in triplicate, and the results were expressed as mmol trolox equivalents (TE)/g sample (dw).

#### 2.7.2. Ferric-Reducing Antioxidant Power (FRAP)

The ferric-reducing power was evaluated as described by Tsai et al. [21], with minor modifications. Briefly, 100 μL of sample was mixed with 100 μL of phosphate buffer (200 mM, pH 6.6) and 100 μL of potassium ferricyanide (1%, *w*/*v*), incubated at 50 °C for 20 min, and centrifuged (200 g, 4 °C, 10 min) after adding 100 μL of TCA (10%, *w*/*v*). A total of 250 μL of the supernatant was mixed with 250 μL of water and 50 μL of ferric chloride (0.1%, *w*/*v*), and the absorbance was measured at 700 nm. BHT was used as positive control. Analyses were performed in triplicate, and the results were expressed as μmol TE/g sample (dw).

#### 2.7.3. ABTS Radical Scavenging Capacity

The ABTS radical scavenging capacity was determined according to Re et al. [22], with some modifications. ABTS (7 mM) was dissolved in potassium persulfate (2.45 mM) and kept in the dark for 12 h, to produce ABTS^•+^. Then, it was diluted with phosphate buffer saline (PBS 50 mM, pH 7.4), until reaching an absorbance of 0.7 at 734 nm. A total of 10 μL of each sample was mixed with 990 μL of ABTS solution, incubated for 6 min, and its absorbance measured at 734 nm. Ascorbic acid was used as positive control. Analyses were performed in triplicate, and the results were expressed as nmol TE/g sample (dw).

### 2.8. Statistical Analysis

One-way analysis of variance (ANOVA) and Fisher’s Least Significant Difference (LSD) tests were performed on the data, using Statgraphics Centurion XVII software (Statgraphics Technologies, Inc., The Plains, VA, USA). The results were expressed as the mean of replicates ± standard deviations, and differences were considered statistically significant at *p* < 0.05.

## 3. Results and Discussion

### 3.1. Physicochemical, Textural, and Rheological Characteristics of Legume Pastes

Legume samples were characterized in terms of moisture content, water activity, pH, and °Brix, which are related directly to stability and microbial safety, as well as color that is associated with organoleptic acceptance and quality perception. The effects of the different cooking methods (ordinary cooking (C), pressure cooking (P), and microwave (M)) on physicochemical parameters of legume pastes prepared from soybean (SO), lentil (LE), and pea (PE) are presented in Table 1. The moisture content values of LE and PE samples were significantly different (*p* < 0.05) according to the method of cooking, showing the highest values for LEP (78.79 %) and PEP (68.60%) within each type of legume paste; however, non-significant differences (*p* < 0.05) were found in SO samples, despite the different cooking. Water activity (a_w_), which refers to the amount of free water available for bacterial growth, presented values of 0.99 for all samples, whereas the pH values of pastes ranged from 5.96 to 7.11, showing significant differences between the applied cooking methods for both SO and LE samples (Table 1). On the other hand, soaking and cooking processes have been widely reported to cause considerable losses in soluble solids, including ANC, sugars, pigments, starch, non-protein nitrogenous compounds, proteins, and especially vitamins and minerals [23,24]. The water temperature, as well as the type of seed and its physicochemical properties, influences the total solid loss in legumes [25]. Different cooking methods extracted total soluble solids (TSS) to various extents, obtaining values ranging between 13.13 and 13.78 °Brix for SO, between 2.77 and 7.80 °Brix for LE, and between 5.85 and 13.49 °Brix for PE pastes, depending on the method of cooking within each legume type (Table 1). In all cases, the amount of TSS was significantly (*p* < 0.05) higher in microwave-cooked legumes than the other cooking methods. Microwave energy would lead to an immediate heating within the product, without changing molecular structure, and, consequently, it might cause both lower destruction of tissue cells and lower leaching out of soluble compounds from seeds into the cooking water, as compared to conventional processes. Nevertheless, previous studies preparing chickpea and lentils by different processing methods suggested that microwave cooking would improve the nutritional quality of legumes by reducing the level of ANC, as well as increasing protein digestibility and retention of vitamins and minerals [24,26].

Color is an important parameter in food quality because it determines consumers’ acceptability. Color attributes, including lightness (L*), chroma (C*), and hue (h*) of the different legume pastes, are shown in Table 1. In SO samples, the highest values of L* and h* were found for SOM while C* did not show significant differences (*p* < 0.05) between samples. Similar results were observed for LE, but the C* was lower for LEC than for LEP. On the other hand, the L* of PE samples was not significantly (*p* < 0.05) affected by the type of cooking, whereas the highest values of C* and h* were obtained for LEP and LEM, and LEC and LEM, respectively. Different factors can affect the color of legumes during processing, for example, browning reactions, pigment degradation, acidity, ascorbic acid oxidation, and metal contamination during cooking [27].

**Figure 1:**
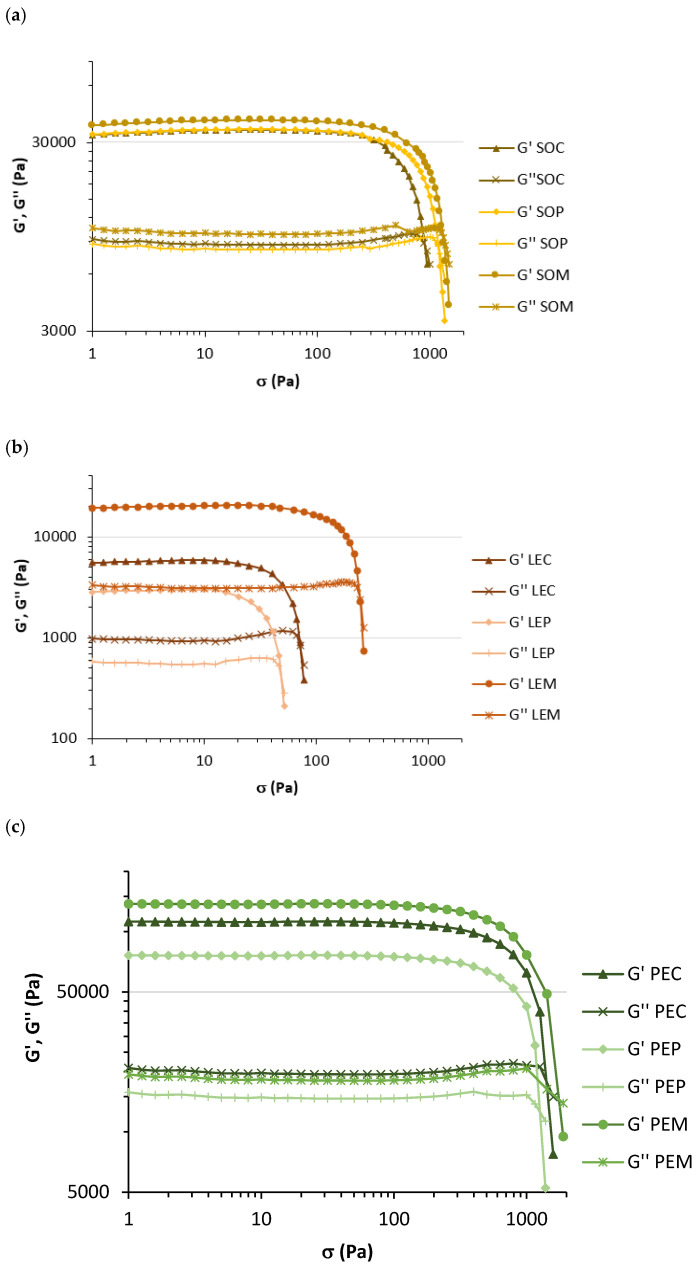
Changes in elastic modulus (G’) and viscous modulus (G’’) according to stress for legume pastes from (**a**) soybean, (**b**) lentil, and (**c**) pea subjected to different cooking methods (SOC, ordinary cooked soybean; SOP, pressure-cooked soybean; SOM, microwave-cooked soybean; LEC, ordinary cooked lentil; LEP, pressure-cooked lentil; LEM, microwave-cooked lentil; PEC, ordinary cooked pea; PEP, pressure-cooked pea; PEM, microwave-cooked pea).

Texture and rheology are useful for physical characterization of samples, and they have an important role not only in determining consumers’ acceptance but also in predicting product stability and designing processing conditions to obtain desired food products. These properties greatly depend on the chemical composition and interaction among food components [28,29]; therefore, microstructural modifications of legume seeds after soaking, cooking, and grinding will determine the rheological behavior of the studied pastes. Textural properties were evaluated by back-extrusion, and the obtained maximum force and AUC values are shown in Table 2. The applied cooking method did not imply significant differences (*p* < 0.05) in the firmness and consistency of SO samples. However, LEM showed higher values than LEC and LEP, and pressure-cooking decreased the firmness and consistency of PE samples. In addition, the viscoelastic behavior of the legume pastes was analyzed, and the results from LAOS and SAOS tests, for characterizing the non-linear and linear viscoelastic properties, respectively, are presented in Table 2. The stress sweep test (LAOS) was used to compare the viscoelastic behavior of food products and establish the limits of LVR, in which the viscoelastic modules are independent of the applied stress. The changes in both modules (G’ and G’’) as stress increases are shown in Figure 1, showing values of G’ higher than G” over the LVR for all the samples. This indicates a low contribution of the viscous component G” to the viscoelastic properties of the samples and thus a typical gel behavior [30]. Alvarez et al. reported that the elastic behavior of chickpea hummus may be mostly attributed to conformational changes, such as gelatinization of starch and coagulation or aggregation of proteins, that occurred during cooking [29]. Additionally, G’_LVR_ and Stress_LVR_ indicated the stiffness and elasticity of the material, respectively. In SO pastes, the different cooking methods did not imply significant differences (*p* < 0.05) in stiffness, but SOC and SOM presented higher elasticity than SOP. LEM showed the greatest stiffness and elasticity among LE pastes, whereas PEP had the least stiff structure and PEC was the most elastic sample. The highest flow point (G’ = G’’), which indicates the breakdown of the internal structure, was found for the legumes subjected to microwave cooking. Furthermore, results obtained from SAOS tests are also shown in Table 2. The different cooking methods did not imply significant differences (*p* < 0.05) in the viscoelastic properties of SO samples, whereas LEM, PEC, and PEM showed the most rigid structure within the respective legume type, with significantly higher G’, G’’, and η* values. These results are in accordance with those obtained by back-extrusion. Values of G’ higher than G’’ and Tan δ values between 0.1 and 1 indicated weak gel properties for all of the legume samples. The predominance of elastic properties is important for palatability and smooth texture, and it may be associated with more promising properties for a pleasant swallowing [31].

### 3.2. Protein Digestibility of Legume Pastes

Processing conditions of legumes, such as soaking, cooking, and grinding, influence protein digestibility and, thus, the availability for intestinal absorption and protein utilization in the diet. Soaking and cooking processes would reduce or inactive ANC, as well as modify the structure of proteins and the matrix of the legumes, whereas grinding of seeds could improve digestibility by breaking up cellular structure and offering a greater surface of contact between the substrate and digestive enzymes [8,32,33]. Thus, the resulted food-matrix structure greatly impacts on protein digestion and the nature of the released peptides, as well as on the structure of the chyme that could limit or modify the action of digestive enzymes.

The effect of cooking methods and GID on protein digestibility of the legume pastes was evaluated by determining total soluble protein contents, TCA-soluble peptides, and free amino groups (Table 3). The content of soluble proteins in soaked, cooked, and ground samples (before digestion) reached values from 3.15 to 15.04 mg/g, with differences between cooking methods. These values suggest a low solubility and, thus, bioavailability of proteins mainly in SOM, LEM, and PEP, within each type of legume, probably due to leaching out of water-soluble proteins into the cooking water. Moreover, proteins may undergo a partial or complete dissociation, denaturation, and even aggregation of unfolded molecules during cooking, resulting in loss of solubility [32,34]. Several studies have evaluated the effect of processing conditions, such as soaking, cooking, germination, or fermentation, on protein digestion of legume seeds [8,17,24,35], showing variations across different and even within the same legume specie, since a variety of parameters, such as temperature, time, moisture content, or particle size, can influence [33]. For instance, mechanical forces applied to soybean samples (particle size produced) and order of treatments (cooking before or after milling) may determine the extent of protein digestion, depending on the fraction of broken cells and then proteins accessible to pancreatic proteases [32,36]. In this sense, the impact of the cell matrix on protein digestibility in each legume would require more in-depth studies.

During GID, the content of soluble proteins significantly decreased (*p* < 0.05) after the oral and gastric stages, in comparison to undigested samples, whereas the amount of peptides and amino acids generally increased (Table 3). Proteolysis begins in the stomach, with the combined action of HCl and pepsin enzyme. HCl leads to an acidic pH that causes proteins to unfold or uncoil due to the rupture of hydrogen and electrostatic bonds, as well as enables the activation of pepsin that breaks down proteins, generating a mixture of peptides of different sizes and free amino acids [37]. However, the action of salivary α-amylase during the oral phase involves the hydrolysis of starch, and no proteolysis was expected to occur. In this regard, the decreased content of soluble proteins but no increase of peptides and amino acids (Table 3) could be due to possible interactions of proteins with polyphenols and/or salivary enzymes, leading to the formation of complexes or protein aggregation [13,38]. It should also be considered that the protein fraction soluble in 5% TCA would be composed of small peptides (< 10 amino acid residues) and free amino acids [39], so larger peptides that are mainly released during the gastric phase were not covered. At the intestinal stage, protein digestion is completed by the action of different proteases and peptidases, mainly trypsin and chymotrypsin enzymes, that further hydrolyze peptide fractions for subsequent absorption [37]. In the present study, the solubility of proteins at the end of GID significantly increased (*p* < 0.05), as compared to previous digestive phases, and a substantial release of small peptides and amino acids was observed for all the legume samples. In fact, the content of soluble peptides reached maximum values ranging from 33.75 to 81.56 mg/g, whereas the content of free amino groups was between 40.13 and 165.28 mg/g, depending upon the legume type and cooking (Table 3). The obtained results evidence an improvement in the nutritional use of legumes during the GID. Similarly, Jamdar et al. reported a decrease in the solubility of proteins after soaking and cooking, but an increase after in vitro digestion, in seven commonly consumed legumes, including lentils and peas [35].

The method of cooking influenced the hydrolysis of proteins and the generation of peptides and amino acids differently for each legume and digestion stage (Table 3). At the end of the GID, the content of soluble proteins was 2.5–3 times higher in SOM than SOC and SOP, whereas non-significant and significant differences (*p* < 0.05) for peptides and free amino groups, respectively, were found between cooking methods. In digested LE samples, the content of proteins and peptides showed a similar trend between cooked samples (LEC ≥ LEP ≥ LEM), but the amount of amino acids was significantly low (*p* < 0.05) for LEP. On the other hand, the cooking method did not significantly influence (*p* < 0.05) the content of soluble proteins in digested PE samples, but PEP showed the highest content of soluble peptides and amino acids. These results indicate that processing conditions modify the structure of proteins and/or the food matrix to a different extent for each case, which would influence the kinetics of protein hydrolysis during GID.

Legumes are a good source of phenolic compounds (PCs) that can play a significant role as antioxidants, as well as establish interactions with proteins, which may modify their bioaccessibility. In this regard, the content of free PCs in the legumes pastes was also evaluated (Table 3), obtaining low values for all the samples before digestion and after the oral phase, but a significant increment (three to six times, depending on the type of legume and cooking) after the gastric stage. This may be attributed to the exposition of water-soluble polyphenols from the legume structure due to the action of pepsin on proteins, as well as the low pH that could enhance the presence of undissociated forms of PCs by reducing ionic interactions, thus promoting their diffusion into the aqueous phase [40]. After the intestinal phase, the content of free PCs significantly decreased, probably because of their degradation or transformation into other compounds due to the mild alkaline conditions in the small intestine [41], or the formation of water-soluble/phenolic micelles occurred in the presence of bile salts and lipids at this stage [40]. PC can interact with proteins by covalent bindings or non-covalent complexes (hydrogen, ionic, or hydrophobic bonding), depending on the size, conformation, and charge of both molecules. These interactions may involve changes in protein structure (complexation, unfolding, or precipitation) or chemical modification of amino acids, which can lead to changes in their properties, such as thermal stability, solubility, digestibility, or antioxidant activity [38,42,43]. What is more, PCs can interact with digestive enzymes such as pepsin and trypsin by forming non-covalent bindings, which will influence their catalytic activity and, thus, nutrient digestibility. The structure of PCs (number and location of hydroxyl groups, glycosylation, and structural complexity), as well as the composition of the enzyme (amino acid composition, molecular size, and structure) and characteristics of the reaction (pH, temperature, and incubation time), would determine these interactions [38,44]. Cooking conditions of legumes, as well as differences in the type and chemical structure of phenolics, can modify bioaccessibility by affecting the hydrophobic non-covalent interactions that may occur between PC and other components of the food matrix, such as proteins or dietary fiber [40]. Finally, it should be noted that many nonphenolic compounds, including proteins and amino acids, among others, show considerable reactivity towards the Folin–Ciocalteu reagent, and, hence, it could cause an overestimation of real polyphenol amounts in high-protein legumes [45]. In view of the results of proteolysis and free PC in the studied legumes (Table 3), it is likely that PCs were bound to proteins mainly at the first stages of GID, but more studies should be carried out to evaluate these possible interactions and their changes during the GID.

### 3.3. Antioxidant Activity of Legume Pastes

The antioxidant activity of the different legume pastes before and during in vitro GID (after oral, gastric, and intestinal phases) were evaluated by DPPH, FRAP, and ABTS methods, since there is no standardized assay available to accurately characterize the overall antioxidant capacity of a sample. These three assays are usually categorized as electron transfer (ET)-based methods, although DPPH and ABTS assays can also act in hydrogen atom transfer (HAT) reactions [46]. The antioxidant activity of legume seeds after processing conditions, such as soaking, germination, fermentation, or cooking, has been widely studied [17,35,47]. However, few studies have evaluated their activity throughout in vitro GID, in which digestive enzymes may hydrolyze proteins and release antioxidant peptides, as well as phenolic compounds bound to protein fractions, thus increasing their availability to interact with reactive oxygen species (ROS).

**Figure 2:**
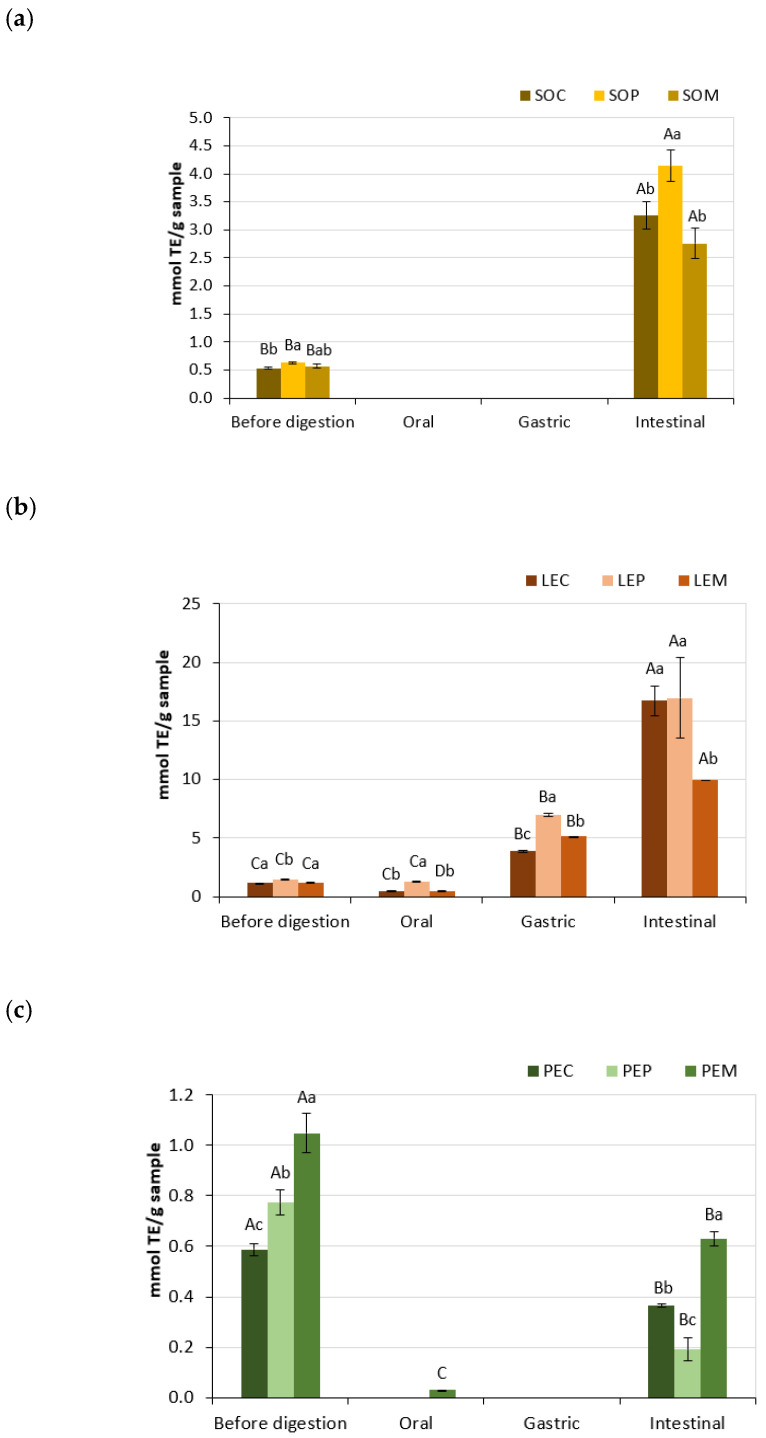
DPPH (2,2-diphenyl-1-picrylhydrazyl) radical scavenging activity of legume pastes from (**a**) soybean, (**b**) lentil, and (**c**) pea subjected to different cooking methods (SOC, ordinary cooked soybean; SOP, pressure-cooked soybean; SOM, microwave-cooked soybean; LEC, ordinary cooked lentil; LEP, pressure-cooked lentil; LEM, microwave-cooked lentil; PEC, ordinary cooked pea; PEP, pressure-cooked pea; PEM, microwave-cooked pea). Capital letter indicates significant differences between phases (before digestion, oral, gastric, and intestinal digestion) within the same legume sample and treatment (*p* < 0.05), whereas lowercase letter indicates significant differences between treated samples (cooking, pressure cooking, and microwave) within the same legume and phase (*p* < 0.05).

Figure 2 shows the results obtained from DPPH assays. In SO and LE samples, the antioxidant activity significantly increased (*p* < 0.05) after GID, reaching values up to 17 mmol TE/g in LEC and LEP. However, the DPPH activity of digested PE samples significantly decreased (*p* < 0.05) in comparison to undigested ones, with the maximum reduction (by four-fold) for PEP. It was noted that LE samples showed a continuous increase of antioxidant activity throughout GID, but no DPPH activity was found in SO and PE samples after the oral and gastric stages. Dawidowicz et al. reported that the food matrix and several factors, such as the solvent of the reaction, water content, and hydrogen ion concentration, could have an influence on the amount of unreacted DPPH^•^ and, thus, on the estimation of antioxidant activity [48]. Moreover, many antioxidants may react slowly or even not interact with the DPPH^•^, due to steric inaccessibility, which is a major determinant of the reaction [46]. The action of intestinal enzymes probably enhanced the accessibility of compounds to the radical site of DPPH rather than the gastric pepsin; therefore, the antioxidant activity increased after GID (Figure 2).

**Figure 3:**
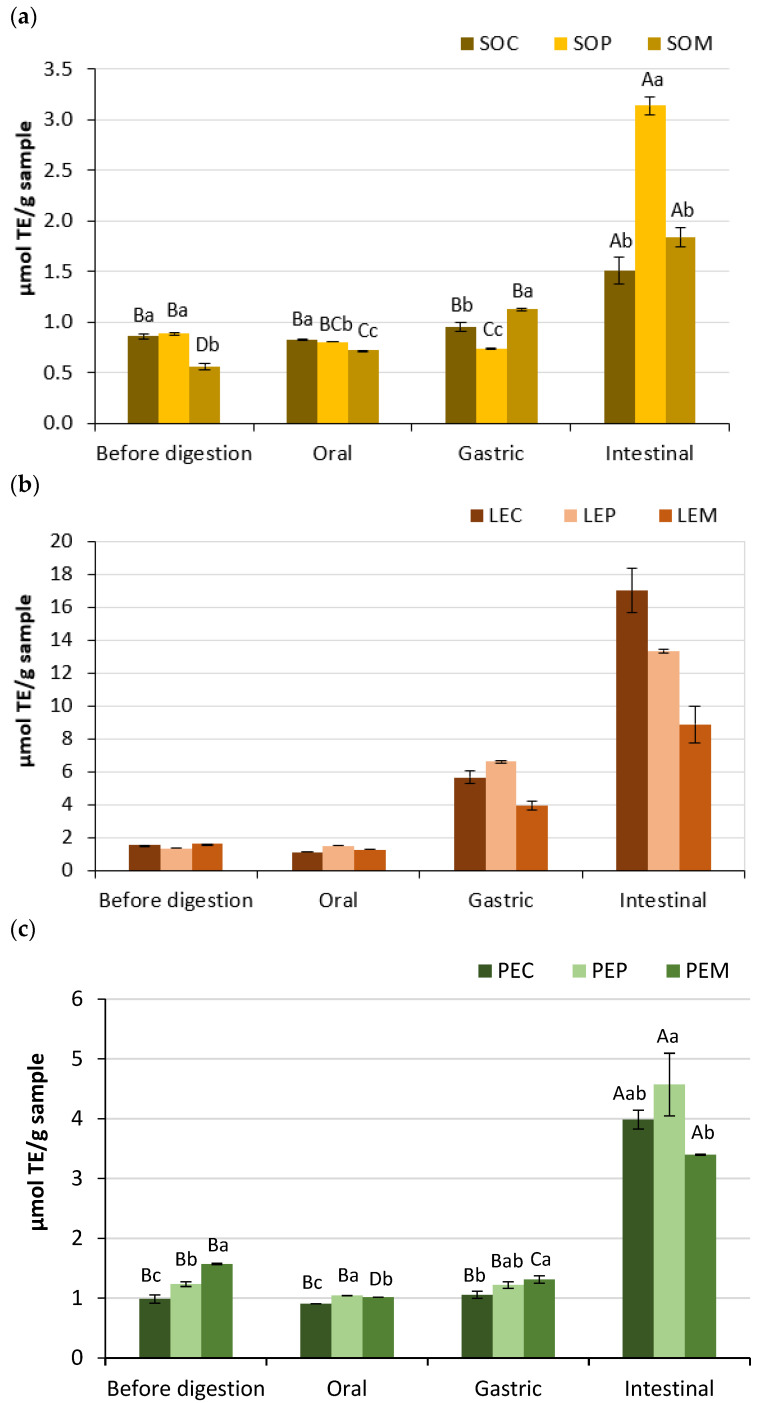
Ferric-reducing antioxidant power of legume pastes from (**a**) soybean, (**b**) lentil, and (**c**) pea subjected to different cooking methods (SOC, ordinary cooked soybean; SOP, pressure-cooked soybean; SOM, microwave-cooked soybean; LEC, ordinary cooked lentil; LEP, pressure-cooked lentil; LEM, microwave-cooked lentil; PEC, ordinary cooked pea; PEP, pressure-cooked pea; PEM, microwave-cooked pea). Capital letter indicates significant differences between phases (before digestion, oral, gastric, and intestinal digestion) within the same legume sample and treatment (*p* < 0.05), whereas lowercase letter indicates significant differences between treated samples (cooking, pressure cooking, and microwave) within the same legume and phase (*p* < 0.05).

Both DPPH and FRAP assays correlate well with the hydrophobicity of the peptides, but the latter is free of the influences from solvent and steric properties of peptides [49]. Results obtained from FRAP assays are shown in Figure 3, showing that all the legume samples showed a significant increase (*p* < 0.05) of the antioxidant activity at the end of the GID, with values ranging from 1.5 to 17 µmol TE/g. This suggest that soluble peptides and free amino acids could be effective electron donors after digestion. A strong increase in FRAP was observed between undigested and digested LE samples (by 5.5–11-fold, depending of the cooking method), which was especially higher than that reported by Jamdar et al. In that study, FRAP values increased by two times after digestion and were attributed to free PC instead of protein-breakdown products [35]. The different processing conditions applied to legumes would lead to differences in the released compounds and antioxidant results. In this sense, the different cooking methods influenced the FRAP activity differently at each stage and legume, with SOP, LEC, PEC, and PEP showing the highest values after the intestinal phase (Figure 3). A similar trend was observed in the DPPH assays of digested samples, except for PE (Figure 2).

**Figure 4:**
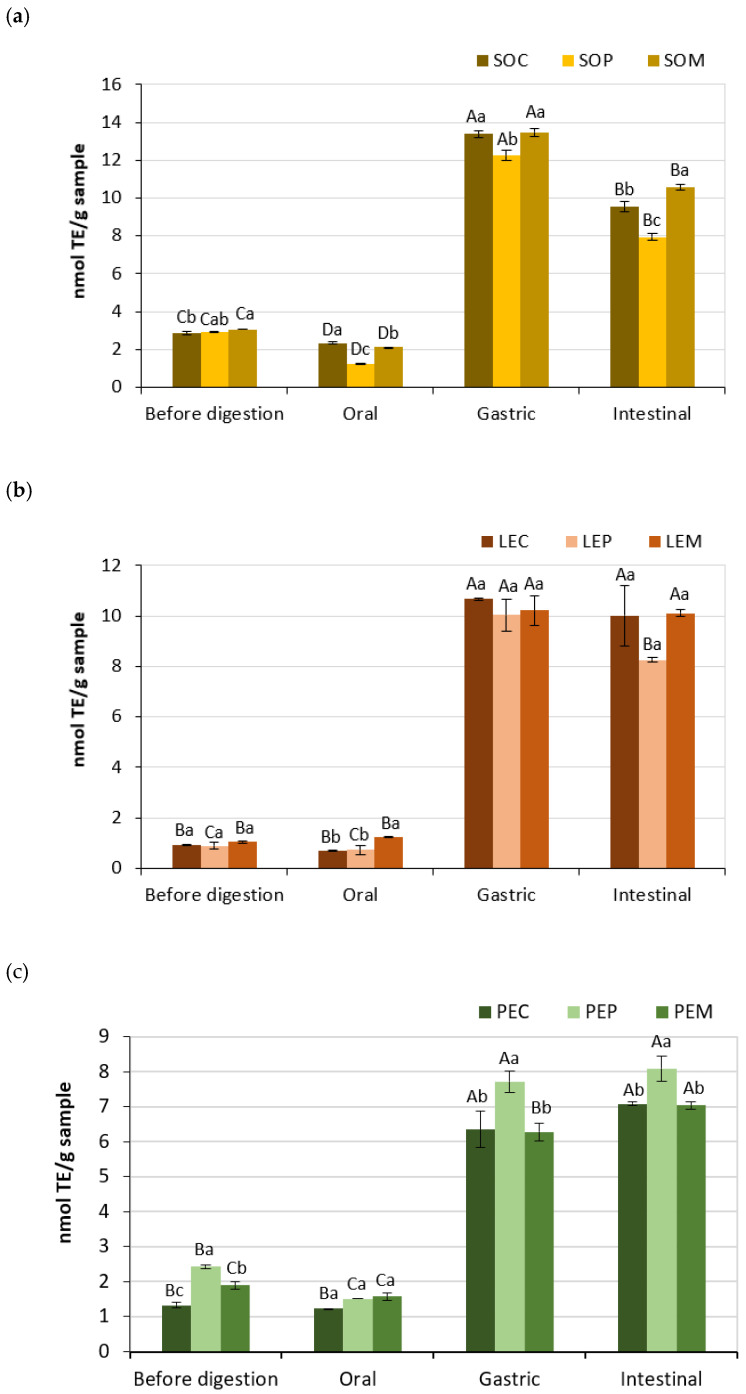
ABTS (2,2′-azino-bis(3-ethylbenzothiazoline-6-sulfonic acid) diammonium salt) radical scavenging capacity of legume pastes from (**a**) soybean, (**b**) lentil, and (**c**) pea subjected to different cooking methods (SOC, ordinary cooked soybean; SOP, pressure-cooked soybean; SOM, microwave-cooked soybean; LEC, ordinary cooked lentil; LEP, pressure-cooked lentil; LEM, microwave-cooked lentil; PEC, ordinary cooked pea; PEP, pressure-cooked pea; PEM, microwave-cooked pea). Capital letter indicates significant differences between phases (before digestion, oral, gastric, and intestinal digestion) within the same legume sample and treatment (*p* < 0.05), whereas lowercase letter indicates significant differences between treated samples (cooking, pressure cooking, and microwave) within the same legume and phase (*p* < 0.05).

The ABTS method is used to evaluate the antioxidant activity of both hydrophilic and lipophilic compounds. Thus, peptides and phenolics present in seed legumes have shown to have ABTS radical scavenging activity [17,35]. The results obtained from ABTS assays of the three legumes are shown in Figure 4. A sharp increase in the antioxidant activity was observed for all the samples after the gastric phase, with the highest increments (more than 10 times) in LE samples. This accords with the large increase of PC previously observed (Table 3), so the antioxidant activity of gastric digesta could be mainly due to phenolics. The action of pepsin could lead to structural changes that may facilitate trapping of free radicals and thus improving quenching by the digested sample. At the end of the GID, the ABTS activity was maintained except for SO samples and LEP, in which it decreased around 20–35%, and PEM samples, in which it slightly increased. At this stage, PC could have been degraded, and thus peptides and amino acids would be mainly responsible for the antioxidant activity. The hydrolysis of the gastric digesta by pancreatin would lead to further peptide-bond cleavages and the accumulation of tripeptides, dipeptides, and free amino acids, which are more hydrophilic than large-size peptides and thus can readily react with the water-soluble ABTS^•+^ [50]. The increment in ABTS radical scavenging activity of digested LE samples as compared to undigested ones (Figure 4b) was especially higher (nearly double) than that reported by Jamdar et al., which was also correlated with the release of free amino groups [35]. When comparing between the different cooking methods within the same legume and GID phase, in general, SOP showed the lowest antioxidant activity, PEP showed the highest values, and the cooking method did not influence significantly (*p* < 0.05) the antioxidant activity in LE samples (Figure 4). Furthermore, it may be pointed out that some authors consider the Folin–Ciocalteu method as a measurement of antioxidant capacity rather than total phenolic content [45,51]. This assay is based on an ET-based reaction that measures the reductive capacity of an antioxidant, and it could show a strong correlation with the ABTS assay [45,46]. In the present study, both assays followed a similar trend in the gastric digesta, but not at the end of the GID.

The action of gastrointestinal enzymes leads to the breakdown of proteins and peptides, the release of amino acids and PC, and the exposition of internal groups, which affect their amount, size, and physicochemical characteristics, thus affecting the antioxidant capacity. The size of peptides is an important factor to determine their ability to cross the intestinal barrier and exert in vivo effects, and, in this regard, most of antioxidant peptides from food proteins have been reported to contain three to six amino acid residues [52]. The protein fraction soluble in 5% TCA would contain peptides lesser than 10 amino acid residues and could include main potential antioxidants. However, amino acid composition and structure, interaction between residues in the peptide sequence, steric and electronic properties, and their solubility in the reaction media would have a key influence on their antioxidant activity [52,53]. Further studies are required to evaluate the structure and characteristics of the peptides, as well as to identify the specific sequences responsible for the antioxidant activity. Moreover, peptide–food-matrix interactions should be also considered, as they can lead to chemical modifications affecting the bioaccessibility and bioavailability of bioactive peptides.

## 4. Conclusions

Legume pastes prepared from SO, LE, and PE seeds subjected to different cooking methods showed differences on their physicochemical properties, as well as on the textural and viscoelastic characteristics, except in the case of SO. All the samples presented a predominance of elastic over viscous properties and had weak gel behavior. Cooking followed by GID improved the protein digestibility and antioxidant activity of the legumes, but the fate and extent at each digestion phase was different according to the legume type and cooking method. Therefore, to establish a conclusion about which specific cooking method is the best for each legume type is not straightforward. More studies are needed to elucidate the effects of processing on food-matrix structure and the interactions occurred between components during GID, as well to identify the bioactive peptides responsible for antioxidant activity. The present study has allowed us to broaden our knowledge about the physicochemical properties, digestibility, and bioactivity of different cooked legume pastes for a future design of protein-rich foods suitable for baby foods or to improve the nutrition of the elderly or the nutrition of people with chewing or swallowing problems.

## Figures and Tables

**Table 1:** Physicochemical parameters of legume pastes from soybean, lentil, and pea subjected to different cooking treatments.

**Parameter**		**Soybean**		
		**Ordinary Cooking**	**Pressure Cooking**	**Microwave Cooking**
**Moisture content (%)**		71.40 ± 1.20 ^a^	70.85 ± 0.37 ^a^	71.15 ± 0.91 ^a^
**aw**		0.99 ± 0.00 ^a^	0.99 ± 0.00 ^a^	0.99 ± 0.00 ^a^
**pH**		6.58 ± 0.01 ^a^	6.50 ± 0.04 ^a^	6.38 ± 0.03 ^b^
**Total soluble solids** **(° Brix)**		13.13 ± 0.09 ^c^	13.27 ± 0.04 ^b^	13.78 ± 1.16 ^a^
**Color**	L*	79.11 ± 0.48 ^b^	79.09 ± 0.16 ^b^	80.42 ± 0.38 ^a^
	C*	20.78 ± 0.68 ^a^	21.32 ± 0.49 ^a^	21.44 ± 0.34 ^a^
	h*	83.23 ± 0.13 ^c^	84.17 ± 0.16 ^b^	87.45 ± 0.35 ^a^
**Parameter**		**Lentil**		
		**Ordinary cooking**	**Pressure cooking**	**Microwave cooking**
**Moisture content (%)**		76.40 ± 0.23 ^b^	78.79 ± 0.43 ^a^	73.57 ± 0.62 ^c^
**aw**		0.99 ± 0.00 ^a^	0.99 ± 0.00 ^a^	0.99 ± 0.00 ^a^
**pH**		7.11 ± 0.04 ^a^	6.98 ± 0.02 ^ab^	6.78 ± 0.12 ^b^
**Total soluble solids** **(° Brix)**		3.48 ± 0.02 ^b^	2.77 ± 0.38 ^b^	7.80 ± 0.33 ^a^
**Color**	L*	48.42 ± 0.47 ^b^	47.24 ± 0.43 ^b^	50.63 ± 0.62 ^a^
	C*	10.94 ± 0.82 ^b^	12. 70 ± 0.02 ^a^	12.16 ± 0.11 ^ab^
	h*	68.19 ± 0.76 ^b^	64.66 ± 1.06 ^c^	77.85 ± 0.03 ^a^
**Parameter**		**Pea**		
		**Ordinary cooking**	**Pressure cooking**	**Microwave cooking**
**Moisture content (%)**		66.56 ± 0.20 ^b^	68.60 ± 0.30 ^a^	65.40 ± 0.07 ^c^
**aw**		0.99 ± 0.00 ^a^	0.99 ± 0.00 ^a^	0.99 ± 0.00 ^a^
**pH**		6.03 ± 0.08 ^a^	5.96 ± 0.09 ^a^	6.06 ± 0.04 ^a^
**Total soluble solids** **(° Brix)**		6.85 ± 1.47 ^b^	5.85 ± 0.14 ^b^	13.49 ± 0.08 ^a^
**Color**	L*	61.33 ± 0.45 ^a^	61.99 ± 0.30 ^a^	62.06 ± 0.21 ^a^
	C*	28.87 ± 0.40 ^b^	29.58 ± 0.33 ^ab^	30.20 ± 0.10 ^a^
	h*	88.31 ± 0.30 ^a^	87.52 ± 0.05 ^b^	88.53 ± 0.13 ^a^

Different letter indicates significant differences between cooked samples within the same legume (*p* < 0.05).

**Table 2:** Texture and viscoelastic properties of legume pastes from soybean, lentil, and pea subjected to different cooking treatments.

**Parameter**		**Soybean**		
		**Ordinary Cooking**	**Pressure Cooking**	**Microwave Cooking**
**Back extrusion**	**Max. force (N)**	20.83 ± 2.87 ^a^	18.79 ± 1.69 ^a^	23.03 ± 3.70 ^a^
	**AUC (N·mm)**	543 ± 50 ^a^	512 ± 57 ^a^	606 ± 100 ^a^
**LAOS**	**G′_LVR_ (Pa)**	39757 ± 8421 ^a^	35077 ± 1051 ^a^	45944 ± 11974 ^a^
	**Stress_LVR_ (Pa)**	158 ± 0 ^a^	115 ± 16 ^b^	158 ± 0 ^a^
	**Flow point (Pa)**	9463 ± 82 ^b^	8495 ± 7 ^c^	9839 ± 128 ^a^
**SAOS**	G* (Pa)	41955 ± 8676 ^a^	36905 ± 1435 ^a^	48645 ± 11802 ^a^
	**G′ (Pa)**	40860 ± 8655 ^a^	35955 ± 1435 ^a^	47490 ± 11922 ^a^
	**G′′ (Pa)**	9489 ± 1105 ^a^	8330 ± 182 ^a^	10445 ± 729 ^a^
	ƞ* (Pa · s)	6678 ± 1381 ^a^	5874 ± 228 ^a^	7743 ± 1877 ^a^
	**δ (°)**	13.20 ± 1.23 ^a^	13.05 ± 0.23 ^a^	12.68 ± 2.24 ^a^
	**Tan δ**	0.23 ± 0.02 ^a^	0.23 ± 0.00 ^a^	0.22 ± 0.04 ^a^
**Parameter**		**Lentil**		
		**Ordinary cooking**	**Pressure cooking**	**Microwave cooking**
**Back extrusion**	**Max. force (N)**	2.16 ± 0.14 ^b^	1.08 ± 0.13 ^b^	7.12 ± 1.58 ^a^
	**AUC (N·mm)**	48 ± 2 ^b^	26 ± 4 ^b^	163 ± 31 ^a^
**LAOS**	**G′_LVR_ (Pa)**	4722 ± 982 ^b^	2869 ± 85 ^b^	24300 ± 7071 ^a^
	**Stress_LVR_ (Pa)**	18 ± 3 ^b^	15 ± 0 ^b^	47 ± 0 ^a^
	**Flow point (Pa)**	735 ± 238 ^b^	487 ± 41 ^b^	3365 ± 306 ^a^
**SAOS**	G* (Pa)	5159 ± 1086 ^b^	3125 ± 90 ^b^	25975 ± 7573 ^a^
	**G′ (Pa)**	5090 ± 1078 ^b^	3073 ± 86 ^b^	25750 ± 7594 ^a^
	**G′′ (Pa)**	839 ± 136 ^b^	569 ± 26 ^b^	3350 ± 316 ^a^
	ƞ* (Pa · s)	821 ± 173 ^b^	497 ± 14 ^b^	4134 ± 1205 ^a^
	**δ (°)**	9.41 ± 0.46 ^a^	10.48 ± 0.19 ^a^	7.63 ± 1.54 ^a^
	**Tan δ**	0.17 ± 0.01 ^a^	0.19 ± 0.00 ^a^	0.13 ± 0.03 ^a^
**Parameter**		**Pea**		
		**Ordinary cooking**	**Pressure cooking**	**Microwave cooking**
**Back extrusion**	**Max. force (N)**	60.59 ± 1.92 ^a^	42.99 ± 5.27 ^b^	66.85 ± 6.80 ^a^
	**AUC (N·mm)**	1696 ± 77 ^a^	1119 ± 98 ^b^	1827 ± 179 ^a^
**LAOS**	**G′_LVR_ (Pa)**	115464 ± 14617 ^ab^	84243 ± 14930 ^b^	132086 ± 1720 ^a^
	**Stress_LVR_ (Pa)**	251 ± 0 ^a^	158 ± 0 ^b^	158 ± 0 ^b^
	**Flow point (Pa)**	21351 ± 239 ^b^	16668 ± 725 ^c^	28959 ± 377 ^a^
**SAOS**	G* (Pa)	124400 ± 15698 ^ab^	87735 ± 14927 ^b^	137082 ± 1671 ^a^
	**G′ (Pa)**	122850 ± 15910 ^ab^	86580 ± 15344 ^b^	135750 ± 1768 ^a^
	**G′′ (Pa)**	19320 ± 523 ^a^	13905 ± 1393 ^b^	18628 ± 441 ^a^
	ƞ* (Pa · s)	19795 ± 2496 ^ab^	13960 ± 2376 ^b^	21815 ± 275 ^a^
	**δ (°)**	9.03 ± 1.40 ^a^	9.34 ± 2.53 ^a^	7.80 ± 0.28 ^a^
	**Tan δ**	0.16 ± 0.02 ^a^	0.16 ± 0.04 ^a^	0.14 ± 0.01 ^a^

Texture parameters represent: maximum force and area under the curve (AUC). Viscoelastic parameters from large amplitude oscillatory shear (LAOS) tests represent: elastic modulus value at LVR (G′_LVR_), stress value at LVR (Stress_LVR_), and flow point. Viscoelastic parameters from small amplitude oscillatory shear (SAOS) tests represent: complex modulus (G*), elastic modulus (G′), viscous modulus (G′′), complex viscosity (ƞ*), phase angle (δ), and loss tangent (Tan δ). Different letter indicates significant differences between cooked samples within the same legume (*p* < 0.05).

**Table 3:** Effect of the cooking method on soluble proteins, peptides, free amino groups and total free phenolics contents of legume samples from soybean, lentil and pea.

		Soybean		
	Digestion Phase	Ordinary Cooking	Pressure Cooking	Microwave Cooking
**Soluble proteins (mg/g)**	Before	15.04 ± 0.62 ^Aa^	12.71 ± 1.48 ^Ab^	9.62 ± 0.55 ^Bc^
	Oral	1.03 ± 0.46 ^Cb^	1.46 ± 0.66 ^Cb^	3.39 ± 0.33 ^Da^
	Gastric	1.48 ± 0.23 ^Cb^	2.75 ± 1.17 ^Cb^	5.88 ± 0.40 ^Ca^
	Intestinal	9.23 ± 1.04 ^Bb^	7.67 ± 0.46 ^Bb^	23.05 ± 2.42 ^Aa^
**TCA-soluble peptides (mg/g)**	Before	8.31 ± 0.25 ^Ca^	8.19 ± 0.16 ^Ca^	7.83 ± 0.02 ^Ca^
	Oral	5.92 ± 0.02 ^Db^	7.87 ± 0.53 ^Ca^	7.94 ± 0.02 ^Ca^
	Gastric	24.36 ± 0.69 ^Bc^	33.45 ± 0.39 ^Bb^	39.63 ± 0.98 ^Ba^
	Intestinal	73.13 ± 0.46 ^Aa^	80.08 ± 3.17 ^Aa^	81.56 ± 5.04 ^Aa^
**Free amino groups (mg/g)**	Before	2.50 ± 0.02 ^Cb^	2.52 ± 0.03 ^Cb^	3.38 ± 0.09 ^Ca^
	Oral	6.00 ± 0.09 ^Ca^	3.82 ± 0.02 ^Cb^	6.00 ± 0.11 ^Ca^
	Gastric	40.41 ± 1.24 ^Bb^	41.78 ± 0.34 ^Bab^	44.02 ± 0.05 ^Ba^
	Intestinal	128.76 ± 5.48 ^Ab^	148.96 ± 13.59 ^Aab^	165.28 ± 10.59 ^Aa^
**Total free phenolics (mg/g)**	Before	1.16 ± 0.02 ^Ba^	1.09 ± 0.02 ^Bb^	1.07 ± 0.01 ^Cb^
	Oral	0.84 ± 0.07 ^Ba^	0.61 ± 0.00 ^Cb^	0.94 ± 0.06 ^Ca^
	Gastric	3.87 ± 0.35 ^Aa^	3.81 ± 0.17 ^Aa^	4.16 ± 0.12 ^Aa^
	Intestinal	—	0.23 ± 0.19 ^Db^	1.63 ± 0.27 ^Ba^
		**Lentil**		
	**Digestion phase**	**Ordinary cooking**	**Pressure cooking**	**Microwave cooking**
**Soluble proteins (mg/g)**	Before	9.09 ± 0.77 ^Aa^	8.71 ± 0.60 ^Aa^	7.17 ± 0.64 ^Ab^
	Oral	1.13 ± 0.10 ^Ba^	1.31 ± 0.15 ^Ca^	1.23 ± 0.09 ^Ca^
	Gastric	0.26 ± 0.14 ^Ba^	0.38 ± 0.13 ^Ca^	0.33 ± 0.19 ^Ca^
	Intestinal	8.87 ± 0.79 ^Aa^	7.33 ± 1.36 ^Ba^	2.83 ± 0.71 ^Bb^
**TCA-soluble peptides (mg/g)**	Before	2.76 ± 0.20 ^Cb^	3.20 ± 0.05 ^Ba^	3.21 ± 0.02 ^Ca^
	Oral	1.35 ± 0.08 ^Db^	1.02 ± 0.04 ^Bc^	1.88 ± 0.04 ^Da^
	Gastric	11.53 ± 0.12 ^Bb^	14.05 ± 0.02 ^Bb^	13.78 ± 0.11 ^Ba^
	Intestinal	77.93 ± 0.28 ^Aa^	52.55 ± 11.22 ^Ab^	41.64 ± 0.25 ^Ab^
**Free amino groups (mg/g)**	Before	2.86 ± 0.05 ^Cb^	3.03 ± 0.02 ^Cb^	3.73 ± 0.08 ^Ca^
	Oral	6.20 ± 0.08 ^Ca^	6.63 ± 0.21 ^Ca^	6.43 ± 0.24 ^Ca^
	Gastric	30.97 ± 0.55 ^Ba^	25.08 ± 0.97 ^Bb^	29.71 ± 0.33 ^Ba^
	Intestinal	74.90 ± 4.55 ^Aa^	40.13 ± 7.00 ^Ab^	73.95 ± 11.56 ^Aa^
**Total free phenolics (mg/g)**	Before	0.56 ± 0.00 ^Cb^	0.64 ± 0.02 ^Ba^	0.63 ± 0.01 ^Ba^
	Oral	0.59 ± 0.05 ^Cb^	0.77 ± 0.01 ^Ba^	0.54 ± 0.04 ^Bb^
	Gastric	3.16 ± 0.01 ^Aa^	3.00 ± 0.22 ^Aa^	2.95 ± 0.21 ^Aa^
	Intestinal	2.21 ± 0.60 ^Ba^	—	—
		**Pea**		
	**Digestion phase**	**Ordinary cooking**	**Pressure cooking**	**Microwave cooking**
**Soluble proteins (mg/g)**	Before	6.28 ± 0.41 ^Ba^	3.15 ± 0.25 ^Bb^	5.82 ± 0.19 ^Ba^
	Oral	3.76 ± 0.31 ^Ca^	1.18 ± 0.17 ^Cc^	2.23 ± 0.17 ^Cb^
	Gastric	0.79 ± 0.10 ^Da^	0.67 ± 0.02 ^Ca^	0.88 ± 0.24 ^Da^
	Intestinal	8.16 ± 1.04 ^Aa^	9.05 ± 1.31 ^Aa^	9.07 ± 0.79 ^Aa^
**TCA-soluble peptides (mg/g)**	Before	2.58 ± 0.03 ^Cb^	2.27 ± 0.13 ^Cc^	3.79 ± 0.09 ^Ca^
	Oral	1.26 ± 0.05 ^Cb^	2.64 ± 0.11 ^Ca^	2.49 ± 0.14 ^Ca^
	Gastric	5.02 ± 0.42 ^Bc^	11.02 ± 0.72 ^Ba^	7.86 ± 0.41 ^Bb^
	Intestinal	33.75 ± 0.79 ^Ac^	59.76 ± 4.21 ^Aa^	45.05 ± 2.29 ^Ab^
**Free amino groups (mg/g)**	Before	2.06 ± 0.06 ^Dc^	3.61 ± 0.02 ^Ca^	3.26 ± 0.04 ^Db^
	Oral	4.62 ± 0.25 ^Cb^	5.89 ± 0.08 ^Ca^	6.17 ± 0.02 ^Ca^
	Gastric	17.18 ± 0.72 ^Ba^	19.84 ± 2.86 ^Ba^	17.31 ± 0.10 ^Ba^
	Intestinal	61.11 ± 1.19 ^Ab^	74.00 ± 5.47 ^Aa^	51.77 ± 0.76 ^Ab^
**Total free phenolics (mg/g)**	Before	0.59 ± 0.02 ^Bb^	0.71 ± 0.01 ^Ca^	0.71 ± 0.01 ^Ba^
	Oral	0.59 ± 0.04 ^Ba^	0.68 ± 0.00 ^Ca^	0.65 ± 0.05 ^Ba^
	Gastric	1.87 ± 0.09 ^Ab^	2.30 ± 0.08 ^Aa^	1.84 ± 0.05 ^Ab^
	Intestinal	0.31 ± 0.04 ^Cb^	1.15 ± 0.22 ^Ba^	—

Capital letter indicates significant differences between phases (before digestion, oral, gastric and intestinal digestion) within the same legume sample and treatment (*p* < 0.05), whereas lower case letter indicates significant differences between treated samples (cooking, pressure cooking, microwave) within the same legume and phase (*p* < 0.05).
